# Systemic metabolic reprogramming and microbial dysbiosis in Fabry disease: Multi-omics mechanisms and implications for drug development

**DOI:** 10.3389/fphar.2025.1702682

**Published:** 2025-12-18

**Authors:** Nuria Gómez-Cebrián, María Chovi Trull, Elena Gras-Colomer, María Dolores Edo Solsona, José Luis Poveda Andrés, Leonor Puchades-Carrasco

**Affiliations:** 1 Pharmacy Research Group, Instituto de Investigación Sanitaria La Fe, Valencia, Spain; 2 Pharmacy Department, Hospital Universitario y Politécnico La Fe, Valencia, Spain; 3 Dirección General de Farmacia, Generalitat Valenciana, Valencia, Spain; 4 Management Department, Hospital Universitari i Politècnic La Fe, Valencia, Spain

**Keywords:** dysbiosis, Fabry disease, metabolic reprogramming, microbiome, multi-omics, precision medicine

## Abstract

Current treatments, including enzyme replacement and pharmacological chaperones, have improved disease outcomes but often fail to fully prevent progression or alleviate persistent symptoms, underscoring the need for novel therapeutic strategies. Recent systems biology and multi-omics approaches have revealed consistent and previously underappreciated alterations in systemic metabolism and the gut microbiota in FD. Here, we synthesize evidence from metabolomic, lipidomic, transcriptomic, and metagenomic studies in patients and experimental models, highlighting disturbances in redox balance, mitochondrial function, energy metabolism, and microbiota-derived metabolites such as short-chain fatty acids and tryptophan catabolites. These findings point to new mechanisms underlying gastrointestinal, inflammatory, and metabolic complications in FD, with direct implications for biomarker discovery and drug development. We further discuss the challenges of integrating multi-omics data into clinical research, the value of mechanistic studies in disease models, and the potential for translating omics-derived insights into precision diagnostics and targeted therapies. By framing FD as a systemic disorder of metabolic and microbial dysregulation, this review outlines a roadmap for mechanism-based interventions that extend beyond canonical glycosphingolipid targets.

## 1 Introduction

Fabry disease (FD) is a rare X-linked lysosomal storage disorder caused by mutations in the *GLA* gene, which encodes the enzyme α-galactosidase A (α-Gal A). FD has been reported in various ethnic groups (Meikle, 1999), and its estimated prevalence ranges from 1:40,000 to 1:117,000 (Mehta et al., 2004) worldwide, although newborn screening programs have revealed a higher incidence rate, primarily due to the underdiagnosis of late-onset phenotypes (Spada et al., 2006; Hsu and Niu, 2018; Lenders and Brand, 2021; Gragnaniello et al., 2022). Deficient activity of α-Gal A leads to the accumulation of globotriaosylceramide (Gb_3_) and other related glycosphingolipids within the lysosomes of different organs and tissues (Hilz et al., 2018), seriously affecting the kidneys, heart and the cerebrovascular system (Clarke, 2007). Clinically, FD manifestation may present with different features depending on gender, phenotype and severity of the enzymatic deficiency (Arends et al., 2017b). Although both sexes can be affected, clinical symptoms are more severe in males than in their female counterparts, mainly due to the random inactivation of the X-chromosome (Deegan, 2005). Based on the phenotype, the disease can be divided into two subtypes: i) a classical or early-onset phenotype, that is most frequently observed in men exhibiting minimal or absent α-Gal A activity, and ii) a non-classical or late-onset phenotype characterized by residual enzymatic activity and lower Gb_3_ levels compared to the classical phenotype (Smid et al., 2015). Initial symptoms of the early-onset phenotype usually arise during childhood and adolescence and typically include neuropathic pain and gastrointestinal problems (e.g., diarrhea, constipation, abdominal pain). These early manifestations may progressively lead to renal, cardiac and cerebrovascular complications (Oliveira and Ferreira, 2019). A larger group of patients has later-onset phenotypes with varying levels of residual α-Gal A activity, age of onset, and manifestations (Smid et al., 2015).

Diagnosis in males with classical FD typically begins with the measurement of α-Gal A activity in plasma, leukocytes or dried blood spot samples (Desnick et al., 1973; Caudron et al., 2015) followed by genetic testing for confirmation. By contrast, individuals with the later-onset phenotype exhibit higher residual enzymatic activity and can be more difficult to diagnose (Michaud et al., 2020; Lenders and Brand, 2021). In women, due to the variable X-inactivation, diagnosis cannot fully depend on enzymatic activity testing, therefore genotyping is mandatory to confirm the disease (Schirinzi et al., 2008). In addition, elevated levels of Gb_3_ and of its deacetylated derivative globotriaosylsphingosine (lyso-Gb_3_) in serum and urine samples are also useful for the initial diagnosis. Several studies have reported lyso-Gb_3_ as a better biomarker than Gb_3_. In fact, lyso-Gb_3_ is the only widely used biomarker for FD in clinical practice (Burlina et al., 2023), and its plasma levels have been shown to partially correlate with disease severity (Arends et al., 2017a; Nowak et al., 2018). However, it has been associated with false negatives, particularly in female and patients with the late-onset phenotype (Aerts et al., 2008; Talbot et al., 2017). Moreover, it does not clearly correlate with disease progression and has not been formally validated for treatment monitoring (Bichet et al., 2021). Although Gb_3_ and lyso-Gb_3_ are well-characterized biomarkers for FD, increasing evidence suggests that the disease is associated with broader alterations in systemic metabolism and cellular homeostasis (Rocchetti et al., 2022; Gambardella et al., 2024; Biddeci et al., 2025), underscoring that metabolic alterations in FD extend beyond Gb3 accumulation. Importantly, this systemic metabolic imbalance has been proposed to influence gut microbiota composition (Aguilera-Correa et al., 2019). Recent studies link gut dysbiosis to gastrointestinal manifestations, systemic inflammation, and possibly neurological manifestations, suggesting a complex multisystem pathophysiology that current biomarkers fail to fully capture (Aguilera-Correa et al., 2019; Sanchez-Niño et al., 2020; Delprete et al., 2023).

From a clinical management perspective, enzyme replacement therapy (ERT) with recombinant human α-Gal A is the gold-standard therapeutical option, though other available treatments include oral chaperone therapy and conventional adjuvant treatments to manage FD symptoms. Although ERT has substantially improved disease management and quality of life (Hoffmann et al., 2007; Arends et al., 2015; Lee et al., 2022), it does not fully prevent disease progression, particularly in advanced stages. This therapeutic limitation further reinforces the growing interest in elucidating additional pathogenic mechanisms beyond glycosphingolipid accumulation (Ko et al., 2016; Bertoldi et al., 2023; Ozer et al., 2024; Marín Gómez and López-Garrido, 2025).

**FIGURE 1:**
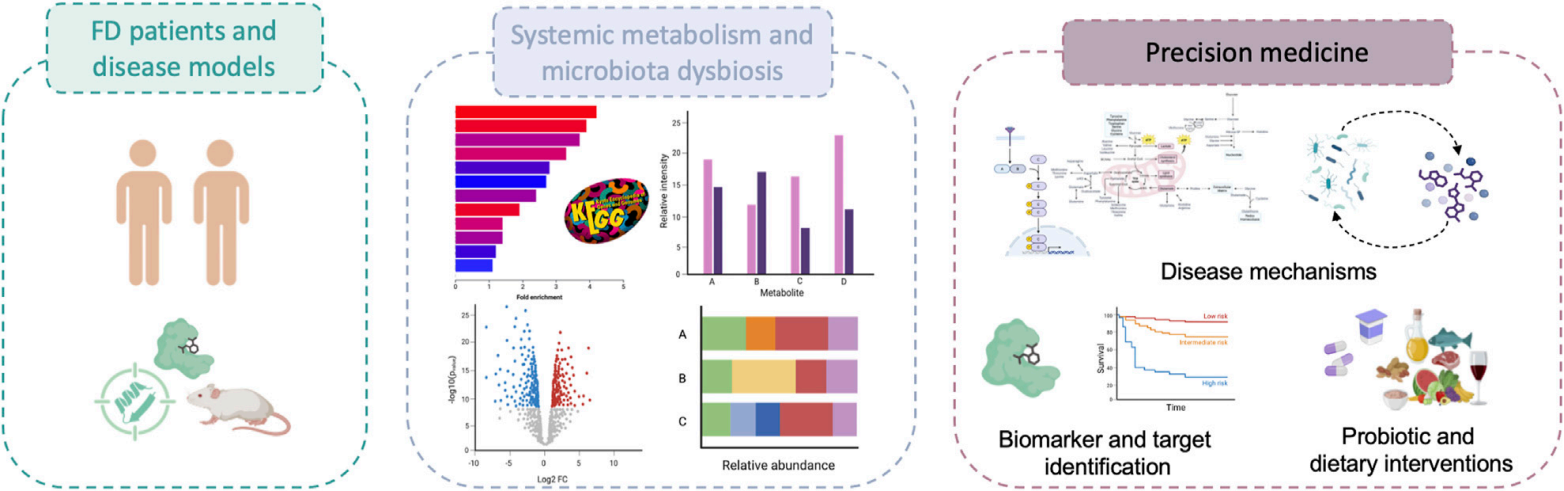
Clinical potential of investigating metabolic and microbiota-related alterations in Fabry disease.

Collectively, these findings indicate that FD is increasingly recognized as a disease of systemic metabolic and immunological disruption, rather than solely as a lysosomal storage disorder. The integration of multi-omic data holds promise for the development of precision medicine tools aimed at early diagnosis, refined patient stratification, and novel therapeutic strategies. As summarized in Figure 1, this evolving perspective calls for a re-evaluation of disease mechanisms and clinical management in FD.

This review aims to critically synthesize current evidence on systemic metabolic reprogramming and gut microbiota alterations in FD, published between January 2010 and June 2025, with a focus on their pathophysiological relevance, potential utility as diagnostic and prognostic biomarkers, and implications for emerging therapeutic approaches. A comprehensive literature search was conducted on Pubmed and Web of Science using the following terms: (“Fabry disease” OR “globotriaosylsphingosine” OR “lyso-gb3”) AND (“metabolism” OR “metabolome” OR “metabolic phenotype”) NOT review; (“Fabry disease” OR “globotriaosylsphingosine” OR “lyso-gb3”) AND (“microbiota” OR “gut alterations” OR “dysbiosis”) NOT review. Only original, full-text articles written in English were included. Titles and abstracts were screened to identify studies applying multi-omics approaches and reporting transcriptomic, lipidomic, proteomic, metabolomic, or microbiome data. Full texts of eligible publications were then reviewed in detail, and relevant methodological and experimental information—including study design, sample type, analytical platforms, and key findings—was systematically extracted. In addition, the terms “Fabry disease,” “metabolism,” and “microbiota” were searched in ClinicalTrials.gov to identify ongoing or completed clinical studies investigating metabolic or microbiome alterations in FD.

## 2 Classical glycosphingolipid biomarkers: Gb_3_, Lyso-Gb_3_, and structural analogs

In FD, Gb_3_ and lyso-Gb_3_ are widely used as diagnostic biomarkers. However, their known limitations in sensitivity and specificity—particularly in female patients and patients receiving ERT—have prompted intensive efforts to identify more sensitive and specific alternatives. Since 2012, metabolomics has emerged as a powerful tool to further characterize the glycosphingolipid profile of FD patients. Through mass spectrometry–based platforms, researchers have identified a variety of structural analogs and related metabolites that may complement or outperform classical markers in terms of diagnostic accuracy and treatment monitoring (Table 1).

**TABLE 1:** Metabolomics studies focused on canonical and analog glycosphingolipids directly associated with α-Gal A deficiency in Fabry disease FD.

Study	Study design	Sample type	Analytical platform	Major findings
Auray-Blais et al. (2012)	59 control vs. 63 untreated FD (M/F)	Urine	UPLC–ESI-TOF-MS	↑ lyso-Gb_3_ and seven analogues (m/z 758, 774, 784, 800, 802, 820, 836)
Auray-Blais and Boutin (2012)	16 control vs. 16 untreated FD (M)	Urine	UPLC–ESI-TOF-MS	↑ 15 isoforms/analogs of Gb_3_
Boutin and Auray-Blais (2015)	16 control vs. 16 untreated FD (M)	Urine	UPLC–ESI-TOF-MS	↑ 22 isoforms/analogs of Ga_2_
Dupon et al. (2013)	34 control vs. 114 FD (42 untreated; 72 treated; M/F)	Plasma	UPLC-Q-TOF-MS	↑ lyso-Gb_3_ and three analogues of lyso-Gb_3_ (m/z 784, 802, and 820); ↓ levels after ERT.
Manwaring et al. (2013)	8 control vs. 12 untreated FD (M)	Plasma	UPLC–ESI-TOF-MS	↑ 24 Gb_3_ analogs

ERT, enzyme replacement therapy; F, female; FD, fabry disease; Ga_2_, galabiosylceramide; Gb_3_, globotriaosylceramide; lyso-Gb3, globotriaosylsphingosine; M, male; UPLC–ESI-TOF-MS, Ultraperformance Liquid Chromatography-Electrospray Ionization-Time-Of-Flight Mass Spectrometry; UPLC-Q-TOF-MS, Ultra-Performance Liquid Chromatography-Quadrupole Time-of-Flight Mass Spectrometry.

Auray-Blais et al. (2012) were the first to report the presence of multiple lyso-Gb_3_ analogs in urine samples from untreated FD patients using targeted UPLC–ESI-TOF-MS. Seven analogs with modified sphingosine moieties (m/z 758, 774, 784, 800, 802, 820, and 836) were detected in both male and female FD patients but were largely absent in controls. Subsequent work by Dupon et al. (2013) expanded these findings to plasma, identifying three additional lyso-Gb_3_ analogs (m/z 786, 802, and 820), as well as lyso-Gb_3_ (m/z 784), previously reported as lyso-ene-Gb_3_ (Auray-Blais et al., 2010). Importantly, many analogs showed higher relative concentrations than lyso-Gb_3_ itself, and some decreased significantly following ERT, suggesting their potential role in both diagnosis and treatment monitoring. These findings suggested that lyso-Gb_3_ analogs may provide enhanced sensitivity, particularly in settings where classical biomarkers fail.

In a different study, focused on male Fabry patients, Auray-Blais and Boutin (2012) were able to identify 15 urinary Gb_3_ isoforms/analogs, grouped into five structural categories: saturated fatty acids (*n* = 6), methylated analogs (*n* = 3), mono-unsaturated species (*n* = 4), a hydrated sphingosine analog, and a di-unsaturated analog. This was the first report of methylated Gb_3_ species, which may represent intermediates in the conversion to lyso-Gb_3_. Similarly, Manwaring et al. (2013) identified 24 Gb_3_ analogs in plasma samples from FD male patients, including five newly proposed biomarkers with strong discriminatory power (AUC 0.989–1 in males). These included short-chain and methylated species, although their diagnostic performance was more variable in females (AUC 0.633–0.883), underscoring the challenges in female diagnosis.

In addition to Gb_3_-related species, the same group explored galabiosylceramide (Ga_2_) analogs in urine, identifying 22 distinct isoforms across seven structural categories (Boutin and Auray-Blais, 2015). Gb_3_ isoforms containing saturated fatty acids were the most abundant (60.9%) compared with 26.3% for Ga_2_. Interestingly, Ga_2_ analogs exhibited a higher proportion of hydroxylated fatty acids, suggesting distinct biosynthetic or degradation pathways for Gb_3_ and Ga_2_ metabolites.

Building on these discoveries, Boutin and Auray-Blais validated the clinical utility of lyso-Gb_3_ analogs using multiplex MS/MS in large cohorts of adult (Lavoie et al., 2013; Boutin and Auray-Blais, 2014) and pediatric (Auray-Blais et al., 2015) FD patients. In these studies, simultaneous quantification of lyso-Gb_3_ and previously identified analogs in plasma, urine, and dried blood spots (DBS) were carried out. In adults, most analogs were undetectable in healthy controls but elevated in FD patients, with significant decreases post-ERT—particularly in males. In children, lyso-Gb_3_ (+16) showed excellent performance in females (AUC = 0.991), while other analogs performed best in males (AUC = 1). In these studies, DBS-based quantification proved consistent with plasma measurements, supporting the potential utility of this approach in clinical settings (Boutin et al., 2023). These age- and sex-specific patterns emphasize the need to tailor biomarker use by patient subgroup in FD.

## 3 Systemic metabolic reprogramming in Fabry disease: insights from human metabolomic and lipidomic studies

Beyond glycosphingolipid profiling, recent metabolomic and lipidomic investigations have revealed a much broader landscape of systemic metabolic dysregulation in FD. These analyses, performed on diverse sample types including plasma, urine, and patient-derived cells, highlight widespread alterations in lipid metabolism, mitochondrial function, redox balance, and energy substrate utilization. Together, these findings suggest that α-Gal A deficiency leads not only to glycosphingolipid storage, but also to profound rewiring of cellular metabolism, with potential diagnostic and therapeutic implications. The following sections summarize key metabolomic and transcriptomic studies that have contributed to this expanded view of FD pathophysiology.

### 3.1 Lipidomic signatures in Fabry disease beyond canonical glycosphingolipids

Recent lipidomic studies have identified a broader spectrum of lipid alterations beyond canonical glycosphingolipids such as Gb_3_ and lyso-Gb_3_. These findings include alterations in sphingomyelins, ceramides, phospholipids, acylcarnitines, and gangliosides, suggesting a broader disruption of lipid homeostasis (Table 2).

**TABLE 2:** Summary of lipidomic studies exploring non-canonical lipid alterations in FD human samples.

Study	Study design	Sample type	Analytical platform	Major findings
Heywood et al. (2019)	58/16 (plasma/urine) control vs. 63 FD (18 asymptomatic; 45 symptomatic)	Plasma, urine	UPLC-MS/MS	↑ long chain CDH and CHM, G_b4_, Gb_3_, lyso-Gb_3_, lyso-Gb_3_-analogues
Ducatez et al. (2021)	60 control vs. 66 FD (21 untreated; 45 treated)	Plasma	LC-MS	86 differentially expressed metabolites: 62 glycerophospholipids, 8 acylcarnitines, 6 sphingomyelins, 5 amino acids and 5 biogenic amines. A 13-metabolite consensus network: 1 biogenic amine (Methionine sulfoxide), 2 lysophosphatidylcholines and 10 glycerophospholipids
Burla et al. (2024)	14 control vs. 11 treated FD	Plasma, platelets	LC-MS	Plasma: ↑ Gb_3,_ Ga_2,_ lyso-dihexosylceramides, S1P, GM3 ganglioside molecular species, ceramide ratios and long-chain acylcarnitines Platelets: ↑ lyso-Gb_3_, acylcarnitines, C16:0-sphingolipids and S1P

CDH, ceramide dihexoside; CHM, ceramide monohexoside; FD, fabry disease; Ga_2_, galabiosylceramide; Gb_3_, globotriaosylceramide; Gb_4_, globoside; LC-MS, liquid chromatography mass spectrometry; lyso-Gb3, globotriaosylsphingosine; S1P, Sphingoid Base 1-Phosphates; UPLC–MS/MS, ultraperformance liquid chromatography tandem mass spectrometry.

Heywood et al. (2019) employed liquid chromatography tandem mass spectrometry to analyze the glycosphingolipid profile of plasma and urine samples obtained from FD patients (treated and untreated) and healthy controls. FD patients exhibited markedly elevated levels of glycosphingolipids in both plasma and urine, including ceramide monohexoside (CMH), ceramide dihexoside (CDH), globotriaosylceramide (Gb_3_), and globoside (Gb_4_). The increase in Gb_3_ was more pronounced in males, while in females, a panel of ten long-chain CDH isoforms in urine provided strong discriminatory power between asymptomatic FD patients and healthy controls (AUC = 0.88). These findings emphasize the diagnostic utility of extended glycosphingolipid profiling in urine, particularly for identifying FD in female patients where classical markers may be less reliable.

In this context, Ducatez et al. (2021) applied a network-based targeted metabolic approach to identify plasma metabolic alterations that help to discriminate Fabry patients from healthy individuals. A total of 86 metabolites were found to be dysregulated, including 62 glycerophospholipids, eight acylcarnitines, and six sphingomyelins. Importantly, the authors refined these findings into a 13-metabolite consensus signature (AUC = 0.965–1.000) composed of 10 glycerophospholipids, 2 lysophosphatidylcholines, and the oxidative stress marker methionine sulfoxide (Met-SO). Beyond their excellent diagnostic performance, these results shed light on the role of glycerophospholipid metabolism and redox imbalance in FD pathophysiology. The study also suggested that combining enzyme or substrate reduction therapy with antioxidant approaches could provide clinical benefit, and proposed Met-SO as a candidate biomarker for treatment monitoring. Furthermore, this work underscores the value of integrative omics and systems-level analyses to unravel the genotype–phenotype complexity of FD.

Burla et al. (2024) applied lipidomics to explore changes in plasma and platelet samples of treated FD patients. Sphingadiene (18:2; O2)-containing sphingolipid species, including Gb_3_ and Ga_2_ were significantly increased in FD patients. Also in plasma, the authors reported increased levels of lyso-dihexosylceramides, sphingoid base 1-phosphates (S1P), GM3 ganglioside species, altered ceramide ratios, and elevated long-chain acylcarnitines. In platelets, the lipidome was similarly affected, with accumulation of lyso-Gb_3_, C16:0 sphingolipids, and S1P. Interestingly, Gb_3_ levels in platelets remained unchanged, suggesting a differential compartmentalization of lipid storage.

Together, these studies extend the metabolic landscape of FD beyond canonical glycosphingolipids, highlighting widespread lipidomic disruptions that affect key structural and signaling lipid species. Importantly, such alterations appear to converge with growing evidence of redox imbalance and mitochondrial dysfunction, suggesting a multifaceted metabolic dysregulation that may underlie disease pathophysiology and offer novel therapeutic targets.

### 3.2 Redox imbalance and mitochondrial dysfunction

In addition to profound disturbances in lipid metabolism, mounting evidence supports the presence of sustained redox imbalance and mitochondrial dysfunction in Fabry disease (FD). Metabolomic, transcriptomic, and functional studies in patient-derived cells, organoids, and biofluids have consistently shown increased oxidative stress markers, altered antioxidant defenses, and mitochondrial abnormalities. Together, these findings suggest that α-Gal A deficiency leads to lysosomal dysfunction, triggering downstream oxidative damage and mitochondrial stress that contribute to disease progression. Key studies addressing these mechanisms are summarized in Table 3.

**TABLE 3:** Summary of studies investigating redox/oxidative stress and mitochondrial dysfunction in FD.

Study	Study design	Sample type	Analytical platform	Major findings
Biancini et al. (2016)	48 control vs. 58 FD	Plasma and urine	LC-MS/MS and spectrophotometric assays	↑ Gb_3_, GSH, TBARS, malondialdehyde, nitrate and nitrite, and ↓ GPx
Tseng et al. (2017)	4 NC-EC vs. 4 FD-EC	FD epithelial cells	RNA microarray	↑ ROS and AMPK activity, ↓ SOD2
Kim et al. (2021)	2 control vs. 3 FD organoids	Kidney organoids	RNA microarray and functional assays	↑ Gb_3_, lipid droplets and Ca^2+^; ↓ GSH metabolism and lysosomal biogenesis genes; ↑ ROS levels, oxidative stress and mitochondrial dysfunction

AMPK, AMP-Activated Protein Kinase; FD, fabry disease; FD-EC, Fabry disease induced pluripotent stem cell-derived vascular endothelial-like cells; GSH, reduced glutathione; GPx, Glutathione Peroxidase; Gb_3_, globotriaosylceramide; LC-MS, liquid chromatography mass spectrometry; NC-EC: healthy control induced pluripotent stem cell-derived endothelial cells; ROS, reactive oxygen species; SOD2, Superoxide Dismutase 2; TBARS, thiobarbituric acid reactive substances.

Biancini et al. (2016) were among the first to comprehensively assess oxidative and nitrosative stress markers, glutathione metabolism, and Gb_3_ levels in plasma and urine samples from FD patients before and during long-term ERT. Their analysis revealed significantly elevated urinary Gb_3_ levels and increased concentrations of reduced glutathione (GSH), alongside a marked decrease in glutathione peroxidase (GPx) activity. In addition, FD patients exhibited higher levels of thiobarbituric acid reactive substances (TBARS), malondialdehyde, and nitric oxide metabolites (nitrate/nitrite), indicating enhanced lipid peroxidation and oxidative damage. Notably, several of these markers remained abnormal despite long-term ERT, suggesting persistent redox imbalance and incomplete biochemical correction. These results not only confirmed systemic oxidative stress in FD but also highlighted its persistence despite ERT, suggesting the need for adjunctive therapies targeting redox balance.

Additional evidence for the interplay between glycosphingolipid accumulation, oxidative stress, and mitochondrial dysfunction was provided by Tseng et al. (2017). In this study, the authors examined endothelial cells derived from Fabry patients and found significantly elevated ROS levels accompanied by downregulation of superoxide dismutase 2 (SOD2), a key mitochondrial antioxidant enzyme. The observed suppression of SOD2 directly impaired mitochondrial antioxidant defenses, thereby linking glycosphingolipid accumulation to mitochondrial vulnerability. Transcriptomic microarray analysis revealed that SOD2 suppression was directly linked to Gb_3_ accumulation. To further explore this relationship, they treated human umbilical vein endothelial cells (HUVECs) with exogenous Gb_3_, which led to dose-dependent decreases in SOD2 expression and concomitant activation of AMP-activated protein kinase (AMPK), a sensor of cellular energy stress. This cascade ultimately resulted in endothelial dysfunction, highlighting the pathogenic role of Gb_3_ in redox imbalance and mitochondrial impairment. These results suggest that dysregulated mitochondrial ROS production, driven by impaired antioxidant defenses, may represent a critical contributor to energy metabolism disturbances and vascular complications in FD, and a potential therapeutic target.

Further insights were gained through the work of Kim et al. (2021), who employed kidney organoids derived from induced pluripotent stem cells of FD patients to investigate disease mechanisms in a three-dimensional human model. The study revealed marked accumulation of Gb_3_ and lipid droplets, accompanied by altered calcium homeostasis. Transcriptomic profiling showed a significant downregulation of genes involved in glutathione metabolism and lysosomal biogenesis, consistent with impaired antioxidant defenses and defective lysosomal function. Importantly, recombinant α-Gal A alone was insufficient to normalize redox parameters. However, supplementation with exogenous GSH restored glutathione homeostasis, enhanced GPx activity, reduced apoptosis, and improved glomerular-like structure formation in FD organoids, pointing to antioxidant strategies as promising adjunctive therapies. These results not only underscore a mechanistic link between lysosomal dysfunction, oxidative stress, and mitochondrial impairment in FD but also demonstrate the value of organoid platforms for mechanistic exploration and preclinical therapeutic testing.

Taken together, these studies establish persistent redox imbalance and mitochondrial dysfunction as central features of Fabry pathophysiology. Importantly, the partial rescue achieved by antioxidant supplementation highlights the potential of combined therapeutic strategies that target both lysosomal correction and redox homeostasis.

### 3.3 Altered energy metabolism in Fabry disease

Beyond redox imbalance, emerging evidence points to profound disruptions in cellular energy metabolism in FD, reflecting a broader bioenergetic reprogramming that extends beyond lysosomal dysfunction (Table 4). These changes encompass mitochondrial remodeling, altered substrate preference, and transcriptional rewiring of central metabolic networks. Together, these findings suggest that α-Gal A deficiency drives systemic and tissue-specific metabolic disturbances with potential implications for disease progression and clinical manifestations.

**TABLE 4:** Metabolomic and transcriptomic evidence of altered energy metabolism in FD.

Study	Study design	Sample type	Analytical platform	Major findings
Schumann et al. (2021)	3 control vs. 3 FD	Renal tubular epithelial cells	LC-MS/MS, Seahorse	↑ TCA cycle intermediates (a-ketoglutarate, succinate and citrate), methylcitrate, glutamine, GSGG, CSSC; ↓ lactate, malate, medium- and long-chain acylcarnitines; ↑ secretion of itaconate; ↓ a-ketoglutarate and methylcitrate in medium. ↓ GSH/GSSG and cysteine/CSSC ratios. ↑ PGC-1α and SIRT1
Gambardella et al. (2023)	30 control vs. 58 FD (25 untreated; 33 treated)	Plasma; skeletal muscle; fibroblasts	FIA-FT-ICR, UHPLC-TIMS	↓ acetylcarnitines and FFAs; ↑ triacylglycerols in plasma. In muscle/fibroblasts: ↑ glycolysis (HIF-1α, HK, PDK1, LDH), ↑ lactate; ↓ CD36 and FASN.
Snandoudj et al. (2024)	9 control vs. 9 *GLA*-edited clones	Immortalized human podocytes	RNA-seq	↑ FAO, glycolysis, amino acid, nucleotide, prostaglandin metabolism; ↓ phospholipid metabolism, heparan sulfate degradation

CSSC, cystine disulfide; FAO, fatty acid oxidation; FASN, fatty acid synthase; FD, fabry disease; FIA-FT-ICR, Flow Injection Analysis Coupled with Fourier-Transform Ion Cyclotron Resonance; FFAs, free fatty acids; GLA, α-Galactosidase A; GSH, reduced glutathione; GSSG, oxidized glutathione; HIF-1α, Hypoxia Inducible Factor 1 Subunit Alpha; HK, hexokinase; LC-MS/MS, liquid chromatography tandem mass spectrometry; LDH, lactate dehydrogenase; PDK1, Pyruvate Dehydrogenase Kinase 1; PGC-1α, Peroxisome Proliferator-Activated Receptor Gamma Coactivator 1-Alpha; SIRT1, Sirtuin 1; TCA-tricarboxylic acid; UHPLC-TIMS, Ultra-High Performance Liquid Chromatography Trapped Ion Mobility Spectrometry.

Schumann et al. (2021) profiled the metabolic landscape of a renal tubular epithelial cell model of FD using untargeted LC-MS/MS. FD cells exhibited a marked accumulation of tricarboxylic acid (TCA) cycle intermediates (α-ketoglutarate, succinate, citrate), along with elevated glutamine, oxidized glutathione (GSSG), and cystine disulfide (CSSC). Additionally, increased levels of free carnitine and short-chain acylcarnitines were accompanied by a reduction in medium- and long-chain acylcarnitines, consistent with disrupted mitochondrial β-oxidation and altered fatty acid metabolism. Metabolic flux analysis of the culture medium revealed increased secretion of itaconate and reduced levels of α-ketoglutarate and methylcitrate, suggesting mitochondrial hyperactivation and redox imbalance. Decreased GSH/GSSG and cysteine/CSSC ratios further supported a shift toward oxidative phosphorylation under oxidative stress conditions. At the protein level, FD cells showed increased expression of PGC-1α and SIRT1, indicating the activation of a mitochondrial stress adaptation program, which was functionally corroborated by increased oxygen consumption rate (OCR) and extracellular acidification rate (ECAR).

Complementary data from Gambardella et al. (2023) demonstrated systemic impairment of energy metabolism in FD. Their multi-omic analysis identified a glycolytic reprogramming in FD skeletal muscle and patient-derived and fibroblasts, driven by HIF-1α accumulation and miR-17-mediated inhibition of oxidative metabolism. Increased expression of glycolytic enzymes (HK, PDK1, LDH) and elevated lactate production were observed, alongside decreased levels of lipid metabolism regulators (CD36, FASN), indicating impaired fatty acid utilization. Consistently, plasma profiling revealed lower levels of acetylcarnitines and free fatty acids, together with an accumulation of triacylglycerols—hallmarks of compromised mitochondrial β-oxidation. These changes were present in both treated and untreated patients, suggesting that current ERT does not fully correct metabolic alterations. Altogether, the data point to a systemic shift toward anaerobic glycolysis resembling the Warburg effect, potentially contributing to fatigue, organ dysfunction, and reduced exercise tolerance.

To explore these mechanisms at the transcriptomic level, Snanoudj et al. (2024) used RNA sequencing of immortalized podocytes with CRISPR-mediated *GLA* deletion. Pathway enrichment analyses revealed upregulation of multiple metabolic routes, including fatty acid oxidation, glycolysis, amino acid, nucleotide, prostaglandin, and eicosanoid metabolism. Conversely, genes involved in phospholipid metabolism and glycosaminoglycan degradation (e.g., heparan sulfate) were downregulated. Metabolite prediction based on transcriptomic changes revealed upregulation of 419 and downregulation of 64 metabolites, indicating robust cellular reprogramming.

In summary, these multi-omic studies consistently show that FD involves widespread remodeling of energy metabolism characterized by mitochondrial hyperactivation, impaired substrate utilization, oxidative stress, and transcriptional adaptation. These alterations likely underlie key clinical manifestations and suggest novel therapeutic opportunities targeting bioenergetic pathways. Increasingly, animal models are being used to further delineate the systemic and tissue-specific impact of these metabolic disruptions *in vivo*.

## 4 Insights from animal models: mitochondrial dysfunction and oxidative stress

Animal models deficient in α-Gal A have recently been employed to characterize the systemic metabolic consequences of FD *in vivo*. Using transcriptomic, proteomic, and metabolomic approaches in zebrafish and murine *GLA* knockout (KO) models, these studies consistently reveal mitochondrial dysfunction, redox imbalance, and dysregulated energy and amino acid metabolism (Table 5). These findings expand on observations in human samples, offering mechanistic insights into organ-specific manifestations and identifying potential metabolic biomarkers and therapeutic targets.

**TABLE 5:** Multi-omics studies revealing metabolic and mitochondrial alterations in Fabry disease animal models.

Study	Study design	Sample type	Analytical platform	Major findings
Elsaid et al. (2022)	8 WT vs. 8 *GLA* KO	Renal tissue (zebrafish)	RNA-seq	↑ GSH metabolism, carbon metabolism, amino acid biosynthesis (glycine, serine, threonine), Ca^2+^ signaling; ↓ oxidative phosphorylation, TCA cycle, FA metabolism, BCAA catabolism, drug metabolism
Elsaid et al. (2023)	8 WT vs. 8 *GLA* KO	Renal tissue (zebrafish)	Proteomics	↑ Glycolysis/gluconeogenesis, pyruvate metabolism, glycerolipid metabolism; ↓ SOD2, TCA cycle, BCAA and tryptophan metabolism, pentose phosphate pathway, vitamin B6 and arachidonic acid metabolism
Kim et al. (2024)	7 WT vs. 6–7 *GLA* KO (20w/40w)	Serum and urine (mice)	GC-MS/MS, LC-MS/MS	Serum: ↑ glutamate, aspartate, GSSG; ↓ MTA, isoleucine, nucleosides Urine: ↑ 2-hydroxybutyric acid, 3-hydroxypropionic acid, pyruvic acid, glycolic acid, a-ketoglutaric acid and 4-hycroxyphenylpyruvic acid; ↓ methylated nucleosides and kynurenines
Zhang et al. (2024)	6 treated vs. 6 untreated *GLA* KO	Plasma (mice)	HPLC-MS/MS	mRNA therapy modulated metabolites in arachidonic acid, inositol phosphate, amino acid and glycolysis pathways, restoring metabolic homeostasis

BCAA, branched-chain amino acids; FA, fatty acids; GC-MS/MS, gas chromatography tandem mass spectrometry; GLA, α-Galactosidase A; GSH, reduced glutathione; GSSG, oxidized glutathione; HPLC-MS/MS, High-Performance Liquid Chromatography-Tandem Mass Spectrometry; KO, knock out; LC-MS/MS, liquid chromatography tandem mass spectrometry; MTA, methylthioadenosine; SOD2, superoxide dismutase 2; TCA-tricarboxylic acid; w, week; WT, wild type.

In the study by Elsaid et al. (2022), RNA sequencing of kidneys from gla^−/−^ zebrafish revealed upregulation of metabolic pathways involved in glutathione metabolism, glycolysis, amino acid biosynthesis (notably glycine, serine, and threonine), and calcium signaling. In contrast, key components of oxidative phosphorylation, the TCA cycle, fatty acid metabolism, and branched-chain amino acid (BCAA) degradation were significantly downregulated. These transcriptomic shifts suggest a suppression of mitochondrial oxidative metabolism and a compensatory increase in cytosolic energy pathways.

A follow-up proteomic analysis by the same group (Elsaid et al., 2023) confirmed these findings at the protein level, identifying >500 downregulated and ∼100 upregulated proteins in *GLA* KO zebrafish kidneys. Enrichment analyses revealed increased activity in glycolysis/gluconeogenesis, pyruvate metabolism, and lipid biosynthesis, while enzymes related to the TCA cycle, tryptophan catabolism, the pentose phosphate pathway, vitamin B6, and arachidonic acid metabolism were suppressed. Notably, expression of SOD2, a key mitochondrial antioxidant enzyme, was markedly reduced—paralleling findings in FD patient-derived endothelial cells (Tseng et al., 2017). Together, these results support the presence of mitochondrial dysfunction and metabolic reprogramming toward glycolytic dependence in FD kidneys.

In murine models, Kim et al. (2024) investigated plasma and urine metabolomic profiles in *GLA* KO mice at 20 and 40 weeks. In plasma, 27 metabolites were significantly dysregulated, including increased glutamate, aspartate, and oxidized glutathione (GSSG), and decreased levels of methylthioadenosine (MTA), isoleucine, and several nucleosides. In urine, there was accumulation of metabolites associated with glycolysis and oxidative stress, including 2-hydroxybutyrate, 3-hydroxypropionate, α-ketoglutarate, pyruvate, and 4-hydroxyphenylpyruvate. Pathway analysis highlighted impairments in TCA cycle, pyruvate metabolism, nitric oxide biosynthesis, and kynurenine pathway—all consistent with redox imbalance and energy metabolism dysfunction.

Finally, Zhang et al. (2024) explored the effect of mRNA-based α-galactosidase therapy in *GLA* KO mice. Treated animals exhibited partial normalization of plasma metabolites involved in glycolysis, glutamate and alanine metabolism, and inositol phosphate signaling. In contrast, tryptophan degradation and fructose/mannose metabolism were suppressed, suggesting that mRNA therapy modulates key metabolic pathways and may help restore systemic energy balance in FD.

In summary, animal models of Fabry disease provide robust evidence of widespread metabolic dysregulation, including mitochondrial dysfunction, oxidative stress, and impaired energy metabolism. These *in vivo* findings not only reinforce conclusions from patient-derived cells and biofluids but also underscore the therapeutic potential of targeting metabolic pathways in FD. Building on these insights, recent studies have begun exploring another major modulator of host metabolism and immune function: the gut microbiota.

## 5 Gut microbiota and its metabolic impact in Fabry disease

The gut microbiota, defined as the complex community of microorganisms inhabiting the gastrointestinal tract, plays a fundamental role in the regulation of host metabolism, immune function, and systemic inflammation (Hou et al., 2022). Among its key metabolic outputs are short-chain fatty acids (SCFAs), such as acetate, propionate, and butyrate, which are primarily generated through microbial fermentation of dietary carbohydrates and account for more than 90% of total SCFA production (Ríos-Covián et al., 2016). These metabolites contribute to intestinal epithelial homeostasis, maintenance of gut barrier integrity, and regulation of inflammatory pathways (Wang et al., 2017; Niccolai et al., 2019).

Gastrointestinal symptoms—including bloating, abdominal pain, diarrhea, and constipation—are highly prevalent and often debilitating in FD, although they remain under-recognized in clinical practice. Traditionally attributed to autonomic neuropathy, emerging evidence now suggests that gut dysbiosis, characterized by imbalances in microbiota composition and function, may play a more direct and active role in symptom generation (Hilz et al., 2018; Turroni et al., 2018). In particular, altered SCFA production and shifts in microbial taxa have been implicated as contributing factors in gastrointestinal and potentially systemic manifestations of FD (Table 6).

**TABLE 6:** Gut microbiota alterations and associated metabolic shifts in experimental models of FD.

Study	Study design	Sample type	Analytical platform	Major findings
Aguilera-Correa et al. (2019)	Control vs. lyso-Gb_3_ treatment	Human colon microbiota suspension and bacterial isolates	qPCR, HPLC	↑ *Bacteroides fragilis* biofilm; ↑ *Enterobacteriaceae, Enterococcus, Prevotella, B. fragilis;* ↓ *Akkermansia, Bacteroides, Bifidobacterium, C. leptum, B. coccoides-E. rectale, Lactobacillus;* ↑ succinic acid, ↓ butyric acid and formic acid
Delprete et al. (2023)	WT vs. *GLA* KO mice at 3 ages (*n* = 10/group)	Fecal samples	16S rRNA, HS-SPME GC-MS	↑ *Lachnospiraceae, Porphyromonadaceae*, *Rikenellaceae (Alistipes)*; ↓ *Bacteroidales* S24-7; ↑ SCFA synthesis, tryptophan degradation, GABA and p-cresol production pathways

GABA, gamma-aminobutyric acid; GLA, α-Galactosidase A; HPLC, High-Performance Liquid Chromatography; HS-SPME GC-MS, Headspace solid-phase microextraction coupled to gas chromatography–mass spectrometry; KO, knock out; lyso-Gb3, globotriaosylsphingosine; qPCR, quantitative polymerase chain reaction; rRNA, ribosomal RNA; SCFA, short-chain fatty acids; WT, wild type.

In a foundational study, Aguilera-Correa et al. (2019) evaluated the effect of lyso-Gb_3_ on gut microbial communities. Lyso-Gb_3_ was found to enhance the biofilm-forming capacity of *Bacteroides fragilis* and induced significant changes in microbial composition. Specifically, lyso-Gb_3_ promoted the growth of *Enterobacteriaceae*, *Enterococcus*, *Prevotella*, and *B. fragilis*, while reducing the abundance of health-associated genera including *Akkermansia*, *Bacteroides*, *Bifidobacterium*, *C. leptum*, *B. coccoides-E. rectale*, and *Lactobacillus*. These changes were accompanied by increased levels of succinic acid and reduced concentrations of butyric and formic acid. The loss of butyrate-producing bacteria and the expansion of pro-inflammatory taxa suggest impaired intestinal homeostasis and potential systemic effects. Importantly, a commentary by Sánchez-Niño et al. (2020) contextualized these findings, proposing that the lyso-Gb_3_-induced dysbiosis may not only aggravate gastrointestinal symptoms but also contribute to renal and cardiac manifestations in FD through reduced production of anti-inflammatory and epigenetically active metabolites such as butyrate. These insights suggest that dysbiosis may act as a disease modifier, potentially exacerbating systemic inflammation and organ damage.

More recently, Delprete et al. (2023) performed a longitudinal multi-omics analysis of the gut microbiota in *GLA* knockout (KO) male mice, evaluating changes across three developmental stages. Early compositional and functional dysbiosis of the gut microbiota in *GLA*-deficient mice was characterized by an enrichment of *Lachnospiraceae*, *Porphyromonadaceae*, and *Rikenellaceae* (particularly *Alistipes*) and reduction of Bacteroidales S24-7 (Reichardt et al., 2014; Zhang et al., 2019; Louis and Flint, 2017; Adamberg and Adamberg, 2024; Lin et al., 2024). The total amount of SCFAs gradually increased over time in *GLA*-deficient mice. Functionally, FD mice exhibited increased activity of SCFA biosynthetic pathways, tryptophan catabolism, and GABA synthesis. Of particular interest, *Alistipes*—one of the enriched taxa in *GLA* KO mice—has been implicated in the catabolism of tryptophan into indole derivatives and GABA, with potential effects on host serotonin metabolism and behavioral phenotypes such as anxiety (Jiang et al., 2015; Polansky et al., 2015). FD mice also showed increased production and impaired degradation of p-cresol, suggesting potential accumulation of this harmful metabolite (Pascucci et al., 2020; Bermudez-Martin et al., 2021).

These studies collectively suggest that gut dysbiosis in FD is not merely a consequence of disease progression but may actively modulate gastrointestinal symptoms and systemic metabolic imbalances. In this context, microbiota-targeted interventions have emerged as promising strategies to improve patient outcomes. Dietary modifications such as low-protein diets may benefit FD patients with renal involvement by reducing nitrogenous waste, oxidative stress, and insulin resistance (Bilancio et al., 2019; Ko et al., 2020; Mattson et al., 2023; Ko et al., 2017). Moreover, antioxidant therapies including glutathione or vitamin C supplementation in *GLA*-mutant renal organoids have been shown to mitigate oxidative stress and restore cellular redox balance (Kim et al., 2021; Ebenuwa et al., 2023). Likewise, preliminary evidence from a clinical study by Gugelmo et al. (2023) demonstrated that a low-FODMAP diet significantly alleviated gastrointestinal symptoms—including bloating, indigestion, diarrhea, and constipation—in adult FD patients. These findings reinforce the concept that dysbiosis in FD has clinical relevance and may be therapeutically addressable (Lenders and Brand, 2022).

Taken together, both experimental and clinical studies converge on the notion that gut microbiota alterations in FD are not epiphenomenal, but rather integral to symptomatology and systemic pathophysiology. Changes in microbial composition, reduced beneficial SCFA production, and enhanced generation of pro-inflammatory and neuroactive metabolites may exacerbate gastrointestinal complaints, promote low-grade inflammation, and modulate host metabolism. These insights open promising avenues for the development of microbiota-based diagnostic tools and therapeutic strategies in FD.

## 6 Toward personalized management: multi-omic biomarkers and therapeutic opportunities

The marked heterogeneity in clinical course and therapeutic response in FD underscores the urgent need for personalized management strategies. Advances in metabolomics, lipidomics, transcriptomics, and proteomics have uncovered widespread metabolic disturbances, indicating that α-Gal A deficiency drives systemic reprogramming beyond canonical glycosphingolipid accumulation. Rather than analyzing these omics layers in isolation, their integrative assessment holds promise to refine patient stratification, improve disease monitoring, and guide treatment decisions.

Multi-omic approaches have identified composite biosignatures that outperform traditional single biomarkers such as Gb_3_ or lyso-Gb_3_ in reflecting disease activity and treatment response. Panels incorporating oxidative stress–related metabolites, acylcarnitines, and phospholipid species have demonstrated improved discriminatory power across sexes and clinical phenotypes.

Mechanistic studies in zebrafish, GLA-knockout mice, and patient-derived renal organoids have validated several metabolic and microbiota-related alterations identified in patients. These models not only enable dissection of disease pathways—such as glutathione metabolism, tryptophan degradation, or SCFA synthesis—but also provide versatile platforms for preclinical testing of metabolic or microbiome-targeted interventions.

Initial clinical efforts have tested omics-based endpoints. For example, trial NCT02649660 combined proteomic and metabolomic profiling in a small FD cohort, revealing lipidomic alterations associated with treatment response and highlighting candidate biomarkers—including sphingadiene (18:2; O2)-containing Gb_3_, lyso-Hex2Cer, and possibly S1P, GM3, and ceramide ratios—for potential use in screening, prognosis, and monitoring. By contrast, trial NCT03145779, designed to evaluate phenotypic variability in FD, including microbiome and metabolomics analyses during ERT, was terminated prematurely without publicly available results.

Beyond diagnosis, multi-omic profiling also reveals therapeutic vulnerabilities. Systems-level analyses consistently implicate oxidative stress, mitochondrial dysfunction, and altered lipid metabolism as key drivers of disease progression, paving the way for mechanism-driven and drug-repurposing strategies. Methionine sulfoxide, for instance, has been proposed as a biomarker to guide antioxidant-based interventions alongside ERT. Moreover, pathway-level analyses suggest that modulation of lysosomal and inflammatory cascades may further enhance treatment efficacy. By coupling omics-derived biomarkers with mechanism-oriented therapies, a more tailored and adaptive treatment paradigm for FD becomes achievable.

## 7 Conclusion

**FIGURE 2:**
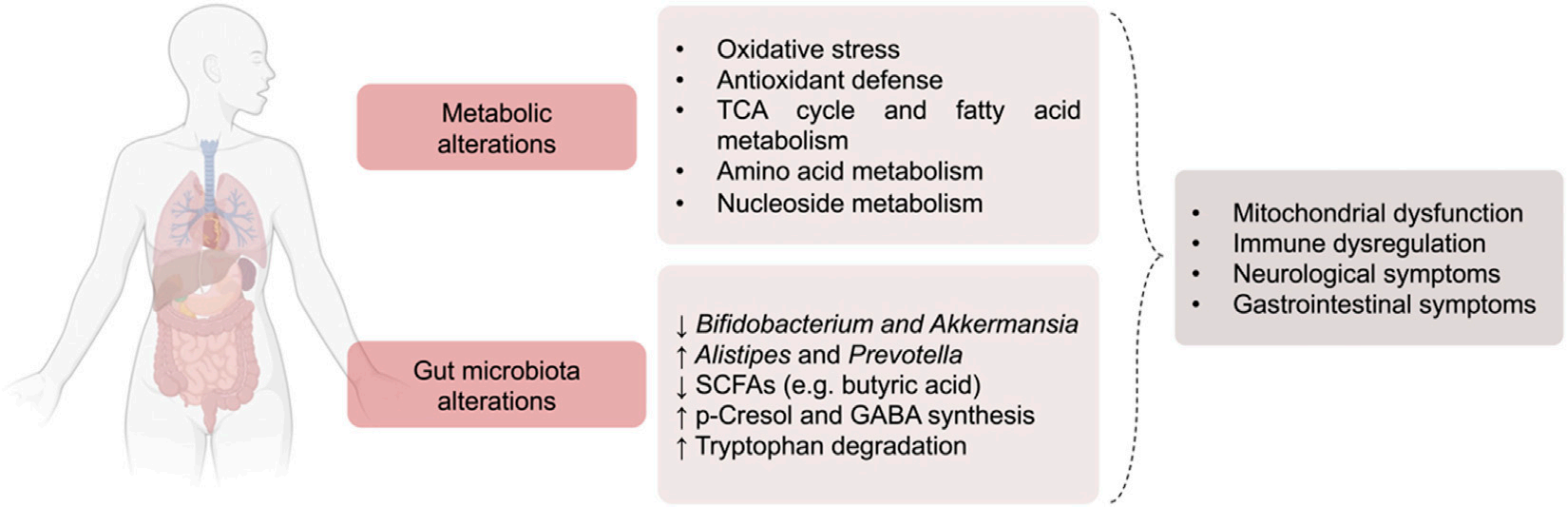
Summary of key systemic metabolic changes and gut microbial alterations described in FD patients and experimental models.

FD is no longer viewed solely as a lysosomal storage disorder caused by α-Gal A deficiency and Gb_3_ accumulation. A growing body of research reveals that FD is a complex, multisystem condition characterized by systemic metabolic reprogramming, mitochondrial dysfunction, oxidative stress, and gut microbiota dysbiosis. These alterations are increasingly linked to clinical features such as gastrointestinal symptoms, immune dysregulation, and potentially neurological involvement. Figure 2 summarizes these systemic alterations across human and experimental studies.

Omics-driven investigations in Fabry patients and animal models have converged to uncover persistent molecular disruptions even in the presence of standard therapies, highlighting the need to expand therapeutic strategies beyond enzyme augmentation. Notably, both metabolomic and metagenomic signatures have shown potential to serve as biomarkers and to inform new therapeutic avenues. However, these findings remain largely confined to research settings and have yet to be fully translated into clinical tools or precision medicine frameworks.

The translation of omics findings into clinical benefit is still at an early stage. Multicenter validation, standardized biobanking, harmonized data integration, and dedicated funding mechanisms are urgently required. Large-scale, longitudinal studies are essential to validate candidate biomarkers across diverse Fabry cohorts. Integrative computational approaches, including artificial intelligence and machine learning, will be critical to harmonize heterogeneous datasets and uncover predictive biosignatures.

Equally important is the continued development of robust experimental models, which are vital to establishing causality and evaluating the clinical relevance of candidate biomarkers or targeted interventions. Integrative omics approaches can inform the rational design of combinatorial therapies, such as coupling ERT with antioxidants, anti-inflammatory agents, or metabolic modulators. Beyond therapy, validated biomarkers could support regulatory approval as surrogate endpoints in clinical trials, expediting the evaluation of innovative treatments.

In summary, embracing the systemic complexity of FD offers a unique opportunity to refine diagnostics, improve disease monitoring, and develop novel adjunctive therapies. Moving toward a mechanism-based, personalized approach—grounded in systems biology and supported by coordinated, multidisciplinary efforts—holds great promise to advance precision medicine and improve the quality of life for patients living with FD.
